# Interleukine-2 serum level in breast cancer patients

**DOI:** 10.22088/cjim.14.3.479

**Published:** 2023

**Authors:** Tutik Harjianti, Andi Fachruddin, Rahmawati Minhajat, Sahyuddin Saleh, Dimas Bayu

**Affiliations:** 1 Division of Hematology and Medical Oncology, Department of Internal Medicine, Medical Faculty Hasanuddin University, South Sulawesi, Indonesia

**Keywords:** Breast cancer, HER-2, ER/PR, Grading, IL-2.

## Abstract

**Background:**

Breast cancer is a form of cancer that typically affects females. In general, cancer is caused by an imbalance between oncogene and supressor gene factors, including immunity factors against cancer cells. This study aims to compare the levels of IL-2 between breast cancer patients and healthy women, and also compare the levels of IL-2 between HER-2 positive and HER-2 negative, ER/PR positive and ER/PR negative, and among different malignancy grades of breast cancer patients.

**Methods:**

This is an observational study using case control method. We include 46 breast cancer patients and 40 healthy women. Blood samples were taken from 46 breast cancer patients (20 HER-2 negative and 26 HER-2 positive patients); 40 of them received hormonal status (29 ER/PR negative and 11 ER/PR positive patients); and from 46 breast cancer patients, 37 of them were divided into malignancy grade. The level of IL-2 was compared between cases and controls and also among the breast cancer patients with HER-2 negative and positive; ER/PR negative and positive; and breast cancer with low, moderate and high grade.

**Results:**

IL-2 level was higher in breast cancer patients than in controls (9.400 pg/mL and 3.990 pg/mL respectively, P=0.003). IL-2 level is significantly higher in the breast cancer cases with positive HER-2 compared to negative HER-2 expression (11.154pg/mL and 7.120pg/mL respectively, P=0.001. No association between ER/PR expression nor breast cancer grading with IL-2 level

**Conclusion:**

IL-2 level is higher in breast cancer patients, especially breast cancer patients with HER-2 positive expression.

Breast cancer is a form of cancer that typically affects females and is heterogeneous in histology, therapeutic response, dissemination patterns to distant sites, and patient outcomes. The incidence is increasing from year to year, both nationally and globally (1). According to Globocan, the incidence of breast cancer in Indonesia has reached to 58.256 cases, or 30.9% out of the total newly diagnosed cancer cases in 2018. Cancer is caused by alterations in cell growth, including increased proliferation, angiogenesis, and apoptosis (2). 

Carcinogenesis is a set of events that occur in phases, beginning with initiation, promotion, and progression to metastatic stages (3). The pathomechanism of breast cancer, like cancer in general, is caused by the interaction of genetic factors and environmental factors. Cancer occurs due to an imbalance between oncogenes and suppressors, including immunity against cancer cells (4). 

Cancer immunity protects the immune system against the onset of cancer. The defense mechanism against cancer cells include the humoral immune system (B lymphocytes) and the cellular immune system (T lymphocytes).Although against tumor cells, cellular immunity plays a main role. Interleukin-2 (IL-2) is a lymphokine generated by Th1 cells that is derived from T lymphocytes (T-helper 1).

Th1 cells control cellular immunity by secreting lymphokines such as IFN- (interferon), TNF-b (tumor necrosis factor), and IL-2, resulting in the activation of macrophages, neutrophils, and CTL (cytolytic cells) (5-7). IL-2-activated neutrophils, macrophages, and NK (natural killer) cells have a role in non-specific immunity against tumor. The effect of cell activation can be cytolytic or cytostatic. This type of immunity does not require antibodies and antigen specificity, so that these cells can kill all types of tumor cells. NK cell proliferation is regulated by T cells: initially, IL-2 promotes NK cell activation proliferation, and secondly, IFN increases IL-2 receptor expression and NK cell progenitor proliferation (6-8). Anatomical pathology of tumor tissue reveals an increase in plasma lymphokine levels and the presence of phagocytic cells, such as macrophages, NK cells, and CTL. This has been demonstrated by prior studies. In the development of active immunotherapy utilizing lymphokine-activated killer (LAK) cells / natural killer (NK) cells, these cells are produced by cultivating lymphocytes with IL-2 and injected to cancer patients (9-11). 

According to Chavey et al., interleukin expression is increased in breast carcinoma than in normal breast including IL-6, IL-8, G-CSF, IFN-γ, MCP-1 and MIP-1β. The most abundant are IL-2, IL-6, IL-8, IL 10, IFN-γ, MCP-1, MIP-1β, TNF-α and to a lesser extent IL-1β and IL-13. Interleukin which shows an increase in breast cancer is inversely correlated with estrogen receptor (ER) and progesterone receptor status. Most cytokines were not correlated with age of cancer diagnosis, tumor size, histologic type or lymph node status. On the other hand, IL-1β, IL-6, IL-8, IL-10, IL-12, MCP-1, MIP-1β increased high grade tumor compared to low grade tumor. In addition, IL-8 and MIP-1β were overexpressed in HER2-positive than in HER2-negative patients (12). This study aimed to compare the levels of IL-2 between breast cancer patients and healthy women, and also compared the levels of IL-2 between HER-2 positive and HER-2 negative, ER/PR positive and ER/PR negative, and among the different malignancy grades of breast cancer patients.

## Methods

This research is an observational study using case-control method. The study was approved by the Ethics Committee of Hasanuddin University Medical Faculty – Dr. Wahidin Sudirohusodo Hospital with entry number No. 0137/H4.8.4.5.31/PP36-KOMETIK/2011.14.Februari 2011. The study was conducted from January 2011 to August 2012. The sample size of this research included 46 breast cancer patients and 40 control healthy women. Among the 46 breast cancer patients, consisted of 26 breast cancer patients with negative HER-2 expression and 20 patients with positive HER-2 expression. From the 46 breast cancer patients, 40 of them received hormonal status (29 patients ER / PR negative and 11 ER / PR positive). HER-2 and ER/PR expression was categorized based on the results of immunohistochemical examinations conducted at the Anatomical Pathology Laboratorium of the Hasanuddin University Medical Faculty. 

The result is defined as positive if the tumor cell membrane is strongly stained, complete in >10% of cells or equivalent to 3+ Herceptest. Otherwise, negative result is defined by weak or moderate appearance of complete cytoplasmic membrane in ≥10% of tumor cells (score 2+) and ≤10% of cells stained focally or only locally from the cytoplasmic membrane (score 1+). From the 46 breast cancer patients, 37 of them is divided into malignancy grades (7 patients with low grade, 16 moderate grade and 14 high grade). The histopathological grading was assessed by an anatomical pathologist and grouped into low, moderate, or high grades based on the tubular formation, nucleus pleomorphism, and mitotic count. The higher the grade is indicates more a malignancy type. 

The examination of IL-2 levels was evaluated from the blood plasma of all breast cancer patients and controls using the immunoassay method. The reagent kit used for human IL-2 examination is a product R&D system, Minneapolis, MN 55413, USA, Cat: D2050, Lot: 298415, ED: 21 Nov 2012. The differences of IL-2 level are analyzed between breast cancer patients and controls, also breast cancer patients with HER-2 positive and HER-2 negative, ER/PR positive and ER/PR negative, and among breast cancer patients with different grades of tumor. The s was used to assess the statistical difference of IL-2 levels between breast cancer patients with HER-2 positive and HER-2 negative. The Mann-Whitney test evaluated the statistical difference in IL-2 levels in breast cancer patients with ER/PR positive and ER/PR negative. And the Kruskal-Wallis test was used to assess the statistical difference in IL-2 levels among breast cancer patients with different grades (low, moderate, and high).

## Results

**Figure 1:**
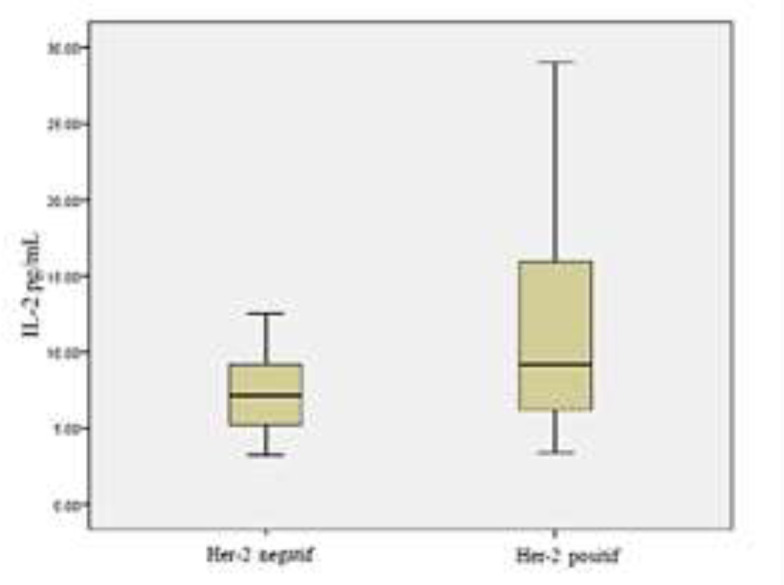
Difference of IL-2 level in breast cancer patients with positive HER-2 and negative HER-2

This study consisted of 46 breast cancer women and 40 healthy women as controls, with a mean age of 47.8 years (median 48.5 years). The IL-2 levels were higher in breast cancer patients (9.400 pg/mL) than in controls (3.990 pg/mL) with a p- value of 0.003 (table 1). This study also evaluated the differences of IL-2 levels in breast cancer patients with positive and negative HER-2 expressions. Table 2 showed a significant difference between IL-2 levels in breast cancer patients with positive HER-2 expression (11.154 pg/mL) and breast cancer patients with negative HER-2 expression (7.120 pg/mL) with a p-value of 0.001. Likewise, it can be seen in figure 1, IL-2 levels were significantly higher in the breast cancer group with positive HER-2 compared to the group with negative HER-2. As seen in table 3, showed the result of IL-2 level among breast cancer patients which was higher in those with ER/PR negative compared to ER/PR positive, but the result was not statistically significant (21,53 pg/mL and 17,77 pg/mL respectively; Mann- Whitney test, P 0,0357). Likewise in table 4, showed the level of IL-2 which was higher in the group with high grade followed by moderate grade and low grade, but the result was not statistically significant (21,57 pg/mL; 19,03 pg/mL; and 13,79 pg/mL respectively; Kruskal-Wallis test, P 0,290). 

**Table 1:** IL-2 level in breast cancer patients and controls

	**Case Control**	**N**	**Mean (pg/mL)**	**Std. Deviation**	P-value*
**IL-2**	**Control**	40	3.9900	2.13959	0.003
	**Case**	46	9.4000	5.94945	

IL-2: Interleukin 2, N: total subjects. *t- test, significant if P< 0.005

**Table 2:** IL-2 level based on HER-2 expression

	**HER-2 expression**	**N**	**Mean**	**Std. Deviation**	P-value*
**IL-2**	**Negative**	20	7.1200	2.33725	0,001
	**Positive**	26	11.1538	7.22519	

HER-2: Human Epidermal Growth Factor Receptor 2, IL-2: Interleukin 2, N: total subjects. *t- test, significant if P< 0.005

**Table 3.** IL-2 level based on ER/PR expression

	**ER/PR expression**	**N**	**Mean**	**Sum**	P-value*
**IL-2**	**Negative**	29	21,53	624,50	0,357
	**Positive**	11	17,77	195,50	

ER/PR: Estrogen Receptor/ Progesteron Reseptor, IL-2: Interleukin 2, N: total subjects *Mann Whitney, significant if P< 0.005

**Table 4:** IL-2 level based on grading of pathology Anatomi

	**Grading**	**N**	**Mean**	P-value*
**IL-2**	**Low Grade**	7	13,79	0,290
	**Moderate Grade**	16	19,03	
	**High Grade**	14	21,57	

IL-2: Interleukin 2, N: total subjects. *Kruskal-Wallis, significant if p< 0.005

## Discussion

The immune system can act as an inhibitor against the initiator of cancer and the development of cancer cells. In this case, the more potent T lymphocyte have anti-tumor receptor cells (6,8). In the review by Yang Y and Lundqvist A, reported the distinct roles of IL-2 and IL-15 in activating various functions in T and NK cells with a particular focus on the signals that participated in the resistance of tumor-derived immune suppressive factors. Furthermore, they concluded the current clinical applications of IL-2 and IL-15 in metastatic malignancies, either as monotherapy or in combination with other agents (13,14). In this study, the IL-2 levels of breast cancer patients were evaluated. The results showed that IL-2 levels in breast cancer patients were significantly higher compared to controls (9.400 pg/mL and 3.990 pg/mL, p = 0.003). It is known that IL-2 is a lymphokine derived from T lymphocytes and is only produced by Th1. Through the secretion of lymphokines such as IFN- (interferon), TNF- (tumor necrosis factor), IL-2, Th1 cells regulate cellular immunity resulting in activation of macrophages, neutrophils, and CTL (cytolytic cells) (4,6). 

Non-specific immunity against tumor is played by neutrophils, macrophages and NK cells which have been activated by IL-2. The effect of cell activation can be cytolytic or cytostatic. This type of immunity does not require antibodies and antigen specificity, so these cells can kill all types of tumor cells. NK cell proliferation is controlled by T cells: initially, IL-2 increases the proliferation of NK cell activation and secondly, IFN increases IL-2 receptor expression and proliferation in NK cell progenitors (6,8). In our study, from 46 breast cancer patients, 26 patients had negative HER-2 expression and 20 patients with positive HER-2 expression, the IL-2 levels were significantly higher in the group with positive HER-2 expression compared to negative ones (11.154 pg/mL and 7.120 pg/mL, P = 0.001). These results are consistent with previous study by Muraro E, et al., wherein the breast cancer group with HER-2 negative, the serum lymphokine profile (IL-2, IL-6, IL-8) was significantly lower compared to controls and the lymphokine profiles (IL-2, IL-1, IL-6, IL-8, IL-10) were significantly lower compared to the group with HER-2 positive (8). 

According to Vignoli A et al., the lower the level of cytokine profile IL-2 and IL-10, the higher the incidence of breast cancer recurrence, altough this study was found statistically not significant (15). Another study by Asgari et al., showed that low levels of IL-2 cytokines were significantly associated with recurrence rate for HER-2(+)/ER(+) breast cancer regardless of the use of adjuvant therapy with tamoxifen and/or aromatase inhibitors. Asgari et al. said that the immune metabolic profile can be used as an independent predictor factor to predict the recurrence of breast cancer, and should be checked prior to any treatment or surgery. Analogously, the cytokine profile provides interesting information by identifying the baseline of IL-2 and IL-10. The lower the baseline, the higher the chance of reccurency (16). Previously, Arduino S. in his study also showed that very low levels of IL-2 were associated with a higher risk of recurrence (17). 

Furthermore, the statistical analysis also verified no significant difference in IL2 levels in ER/PR negative and ER/PR positive (21,53 pg/mL and 17,77 pg/mL; Mann- Whitney test, P: 0,0357). Although statistically there was no significant difference, there was a tendency for negative ER/PR to have higher IL 2 levels than positive ER/PR breast cancer. The study by Chavey C et al., get that negative ER/PR breast cancer group obtaining IL-6, IL-8, G-CSF, IFN-γ, MCP-1 and MIP-1β, IL-2, IL-6, IL-8, IL10, IFN-γ, MCP-1, MIP-1β, TNF-α is overexpressed (12). In particular, Vignoli A et al., in their study found that patients with ER (+) breast cancer not only had an escalation of several T cell triggering factors such as IL12-p70, TNF- α; but also showed an increase level of several cytokines that suppress the work of T-cells such as IL-10, IL-8 and TGF-1 as well as a decrease in both T-cell activating factors (IL-1α and IL-2). The result was found not significant due to high sample variabilities, and small size of the sample (15). 

Thus, low levels of IL-2 and IL-10 found in relapsed ER (+) breast cancer patients reflect a decreased ability to respond quickly to therapy. Both cytokines play a role in modulating the patient's immune system, and are involved in the response to trastuzumab taxanes and hormonal therapy. The higher levels of IL-2 obtained in disease-free patients found to be contributing to a more stable neoadjuvant effect of targeted therapy and chemotherapy by rapidly promoting recruitment and activation of natural killer (NK) and cytotoxic CD8+ T lymphocytes at the tumor site, and enhancing trastuzumab-mediated ADCC (16,18). Both IL-2 and IL-10 were found to increase the anti-tumor cytotoxic activity of CD8+ T cells in vitro and a potential synergistic effect for the two cytokines (19). The last comparison among the breast cancer patients with different malignancy grades show that IL-2 level was higher in the group with high grade, followed by moderate grade and low grade, but the result was not statistically significant (21,57 pg/mL; 19,03 pg/mL and 13,79 pg/mL respectively; Kruskal-Wallis test; P: 0,290).

Lee H identified five genes (TRAT1, IL21R, IGHM, CTLA4 and IL-2RB) that are used in prognostic index. The immune system plays a key role in the regulation of T-cell, B-cell, natural killer cell (NK), and interleukin signaling functions. Lee H found that high expression of those genes was associated with favorable prognoses in a high-risk subtipe group of breast cancer patients (20). According to Wang L.N. et al., IL21R and IL21 contribute in accelerating breast cancer cell migration and invasion (21). Another study conducted by Mittal D et al., demonstrated that higher IL21R expression in HER2+ breast cancer patients was associated with a positive effect of trastuzumab therapy with disease-free improvement (22). This suggests the role of IL21 R as a predictive marker for anti-HER2+.

However, the prognostic significance and the role of these five genes in breast cancer is still uncertain, and it should be noted that the study showed their expression was associated with a favorable prognostic for breast cancer in the high-risk subgroup (20). In clinical studies, Tunon G et al. demonstrated that IL-2 expression, including its three receptor chains, was more prevalent in breast-infiltrating malignancies than in in situ surgical samples (23). The conclusion of this study is that IL-2 level was significantly higher in breast cancer patients, in particular the groups with HER-2 positive expression.

## Acknowledgments

This in accordance with the knowledge that the infiltrating neoplasms are characterized by higher aggressive. We would like thank the study participants and the Deputy of Internal Medicine Research of Hasanuddin University.

### Funding:

This study was conducted without external funding sources.

### Conflict of Interests:

We have no conflict of interest to declare.

### Authors’ contribution:

TH: Conception and design of the article

TH, AF, RM: acquisition of data, analysis and interpretation of data

TH, AF, RM, SS, DB: writing of the article, revision of its content; 

All authors have read and approved the final version of the article before submission.
